# A Case Of Convergence: Why Did a Simple Alternative to Canonical Antibodies Arise in Sharks and Camels?

**DOI:** 10.1371/journal.pbio.1001120

**Published:** 2011-08-02

**Authors:** Martin F. Flajnik, Nick Deschacht, Serge Muyldermans

**Affiliations:** 1 Department of Microbiology and Immunology, University of Maryland at Baltimore, Baltimore, Maryland, United States of America; 2 Laboratory of Cellular and Molecular Immunology, Vrije Universiteit Brussel, Brussels, Belgium; 3 Department of Molecular and Cellular Interactions, VIB, Brussels, Belgium

## Introduction

**Figure 1:**
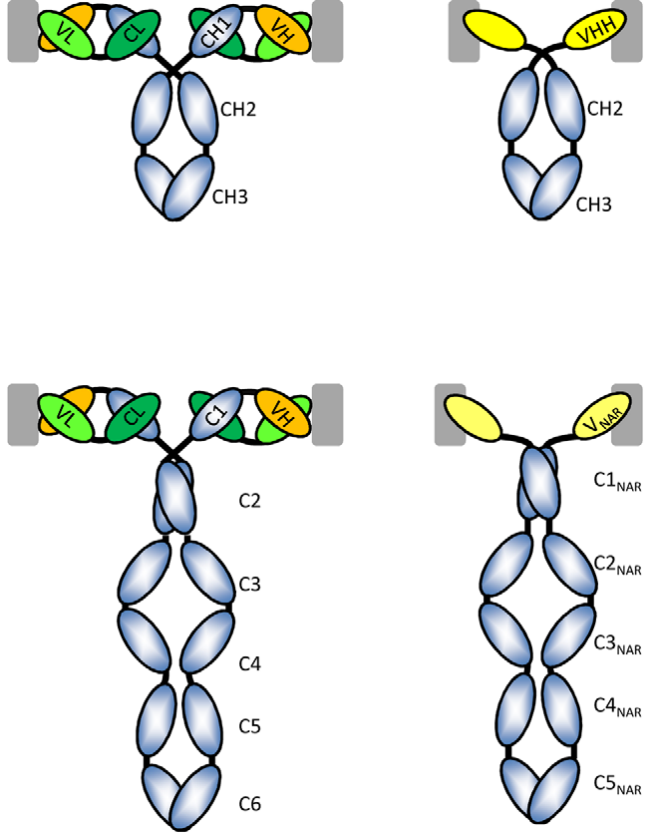
Schematic of classical H_2_L_2_ and H_2_ homodimeric HCAbs. The left column displays the classical antibodies with two identical H-chains and two identical L-chains as they occur in mammals (IgG, top) and cartilaginous fish (IgW, bottom). The L-chain is in green and the antigen-binding site is formed by the paired VH and VL domains. The top right figure is the HCAb as it occurs in sera of camelids, the CH1 domain is missing and there is no L-chain. The antigen-binding site consists of one single domain, known as VHH. The H-chain of the IgW comprises six C domains and a variable domain at the N-terminal end, whereas the IgNAR (bottom right) is a homodimer of a H-chain with five C domains and a V-NAR at its N-terminal end. Note that the equivalent of the first C domain is absent. All of the antibodies are bivalent and the recognition of a possible antigen (gray square) is shown. The VH-VL associated preferentially with flat surfaces on the antigen, whereas the VHH or V-NAR has a preference to interact with cavities on the surface of the antigen.

**Figure 2:**
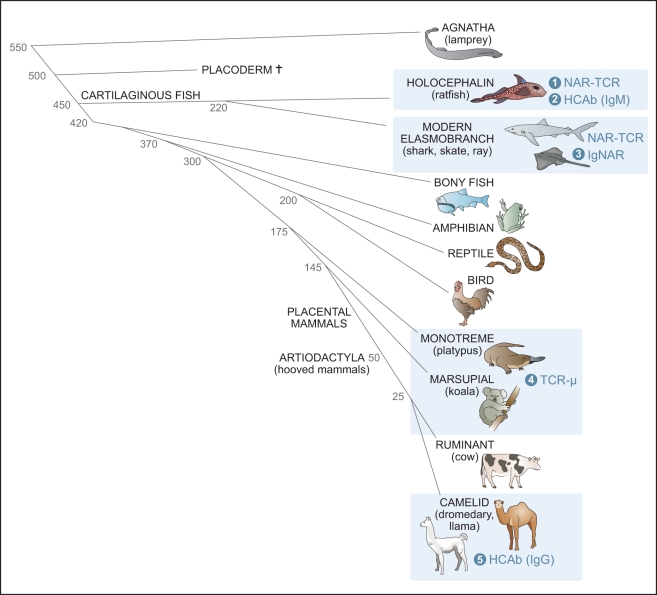
Simplified vertebrate phylogenetic tree emphasizing those taxa possessing single-domain Igs or TCRs. Blue boxes display animal groups shown, or predicted, to have single-domain variable regions. There are five known single domains, at least four of which were derived by convergent evolution, holocephalin HCAb, elasmobranch IgNAR, monotreme/marsupial Igμ, and camelid HCAb. The numbers on the left indicate divergence time (millions of years ago) for the various vertebrate taxa.

The success of our adaptive humoral immune response, mediated by B cells, relies on its capacity to produce multiple antigen-recognition specificities against any possible invader. This antigen-recognition specificity is attributed to antibodies, which display remarkable flexibility and diversity. That diversity is generated by cutting and pasting of immunoglobulin (Ig) gene segments during B-cell development to generate functional variable (V) genes, which are selected from two separate regions of the genome to yield so-called “heavy” (H) and “light” (L) chains (Figure 1; see Section 1 in Text S1). Ig H and L chain V genes have been expanded in various vertebrate taxa to yield multiple families and subfamilies [1]. Given the obvious advantage of the diversity afforded by such multichain antibodies, it was extremely surprising to discover that some species—notably cartilaginous fish (holocephalins, e.g., chimeras, and elasmobranchs, e.g., sharks, skates, and rays) and camelids (e.g., camels and llamas)—developed functional H-chain-only antibodies (HCAbs) (Figures 1 and 2) [2],[3]. Loss of the combinatorial H-L diversification of heterotetrameric (H_2_L_2_) antibodies should result in a handicapped, less diversified antigen-binding repertoire in HCAbs; yet, contrary to expectations, high-affinity, antigen-reactive HCAbs can be elicited to all studied antigens [4],[5]. Even more peculiar, the HCAbs function alongside conventional H_2_L_2_ antibodies. What explains this remarkable evolutionary convergence of functional HCAbs in sharks and camels? What forces drove such distantly related species to produce antibodies with this simple structure? And, if they are so effective why is their prevalence not more widespread in evolution?

## Occurrence of Functional Heavy Chain-Only Antibodies in Camelids and Shark

The antigen-binding fragment of camelid HCAbs consists of a single domain known as a variable domain of the heavy chain of HCAbs (VHH) (Figure 1) [2]. It was demonstrated that HCAbs from dromedaries (one-humped camels) infected with trypanosomes are capable of associating tightly and specifically with these parasite antigens [2]. Subsequently, HCAbs were elicited to many different foreign antigens. HCAbs are present in all living species of the mammalian family *Camelidae*, but not in other ungulates (hoofed mammals). Approximately equal levels of HCAbs and H_2_L_2_ antibodies are present in camel blood [6]. The origin of camelid Ig genes encoding HCAbs is unambiguous. Phylogenetic analyses have demonstrated that the HCAb-dedicated γ genes were derived from γ genes coding for conventional antibodies. The genes emerged and diverged ∼25 million years ago [6],[7], after *Tylopoda* split from other mammals (60–80 million years ago) and before the camel and llama speciation (∼11 million years ago) (Figure 2) [6]. Also, the VHH germline genes descended from the classical variable region of a heavy-chain (VH) genes, and the diversity (D) and JH genes are even shared in the formation of the VH or VHH domains. Thus, the emergence of HCAbs was a relatively recent event in these species.

In the nurse shark (*Ginglymostoma cirratum*), a novel secreted antigen receptor was reported and named the new antigen receptor (NAR) [3]. It too is composed of two covalently associated H-chains with no associated L-chains, but having six domains (Figure 1). Since the molecule shares several functional features with Ig isotypes, it was renamed IgNAR [8], and its N-terminal V domain is known as V-NAR. Later studies showed IgNAR to be induced by immunization with protein antigens with similar kinetics as in a typical IgM response [9]. Although the origin of shark IgNAR is poorly understood, IgNAR is found in all elasmobranchs and thus emerged at least 220 million years ago (Figure 2). However, the single-domain V is also found as a T-cell receptor (TCR) in all living cartilaginous fish (so-called NAR-TCR) [10],[11], showing that this immune system feature appeared at least 350 million years ago (Figure 2). In addition, V-NAR gene clusters actually are present in the TCR-δ loci of all cartilaginous fish and thus it remains uncertain whether they originated as a TCR, an Ig, or perhaps a common antigen receptor ancestor [10].

## Convergent Evolutionary Steps to Generate Functional Heavy Chain-Only Antibodies

Besides the phylogenetic origins, the most significant question regarding molecular convergence is: “What are the critical events or properties that must be introduced to produce a functional HCAb?” We think that three (and perhaps four) crucial requirements must be fulfilled to obtain homodimeric H-chain antibodies: (1) the first constant domain (CH1) within the H-chain that normally serves to anchor the L chain should be absent or extensively modified to prevent its retention within the endoplasmic reticulum (see Section 2 in Text S1) [12]; (2) an extensive and diverse primary repertoire of single V-domains must be generated from which to select the most appropriate binders (see Section 3 in Text S1 on how single V domain sequences are diversified) [6], and the selected autonomous V domain should be able to bind antigen with high-affinity–like conventional antibodies; (3) the V domain must be soluble (i.e., nonsticky) in the absence of a variable region of a light-chain (VL) partner (see Section 4 in Text S1) [13]; and (4) although not absolutely required, noncanonical disulfide bonds should be present that stabilize the single V domain in the absence of VL, and to diversify the repertoire of the antigen binding site (see Section 3 in Text S1) [6],[14],[15].

These requirements underlying the molecular convergence between the HCAbs of camelids and the IgNARs of cartilaginous fish have been met, resulting in the appearance of functional HCAbs that have converged at multiple levels: (1) The structural level: Camelid HCAbs and Ig-NARs are both homodimeric H-chain antibodies devoid of any L-chain. (2) The sequence level: There is a subtle amino acid sequence convergence of the VHH and V-NAR antigen binding domains (i.e., increased frequency of charged and polar residues at the “former VL interface”). (3) The mechanistic level: The strategy whereby H-chain avoids interaction with L-chain (absence of CH1), and the mechanisms that have been employed to create a highly variable antigen-binding repertoire in absence of the H-L combinatorial diversity. (4) The functional level: The VHH of camelid HCAbs and the V-NARs of shark IgNARs target similar epitopes that are distinct from the preferred epitopes of classical H_2_L_2_ antibodies.

## What Drove the Evolution and Selection of Homodimeric H-chain Antibodies?

Such a molecular convergent evolution of homodimeric HCAbs in species as diverse as camels and sharks suggests that the immune systems of their ancestors faced comparable stress, and in order to be selected, propagated, and fixed in the population these molecules likely provided a strong advantage in protecting the animal. Although multiple hypotheses can be proposed, the driving force remains elusive. Possibilities could be an L-chain variant prone to malfunction (by amyloidosis for example [16]), or an L-chain used as a coreceptor for a virus that was B-cell tropic. In such an event, the humoral immune system would benefit from the development of antibodies without L chains. Once HCAbs were designed and widespread in the species, the defective L-chain was no longer expressed; or it was modified leading to alleviation of its malfunctioning; or to prevention of virus recognition so that the viral threat disappeared.

In an alternative and possibly more realistic scenario, HCAbs emerged from the need (or advantage) for the adaptive humoral immune system to develop antibodies with a smaller, prolate (rugby ball-like)–shaped, single-domain, antigen-binding V domain that could complement canonical antibodies with their planar or concave binding sites (see Section 5 in Text S1). Such minimal-sized, single-domain antigen-binding fragments have smaller footprints on the antigen and provide access to cryptic epitopes that are inaccessible to VH/VL-based antibodies, or to epitopes with a concave architecture, such as the catalytic clefts of enzymes [17]. Indeed, from several crystal structures of shark V-NAR and camelid VHH bound to antigen it seems clear that the HCAbs of camelids and sharks are better equipped to target these epitopes [15],[18]–[20].

**Figure 3:**
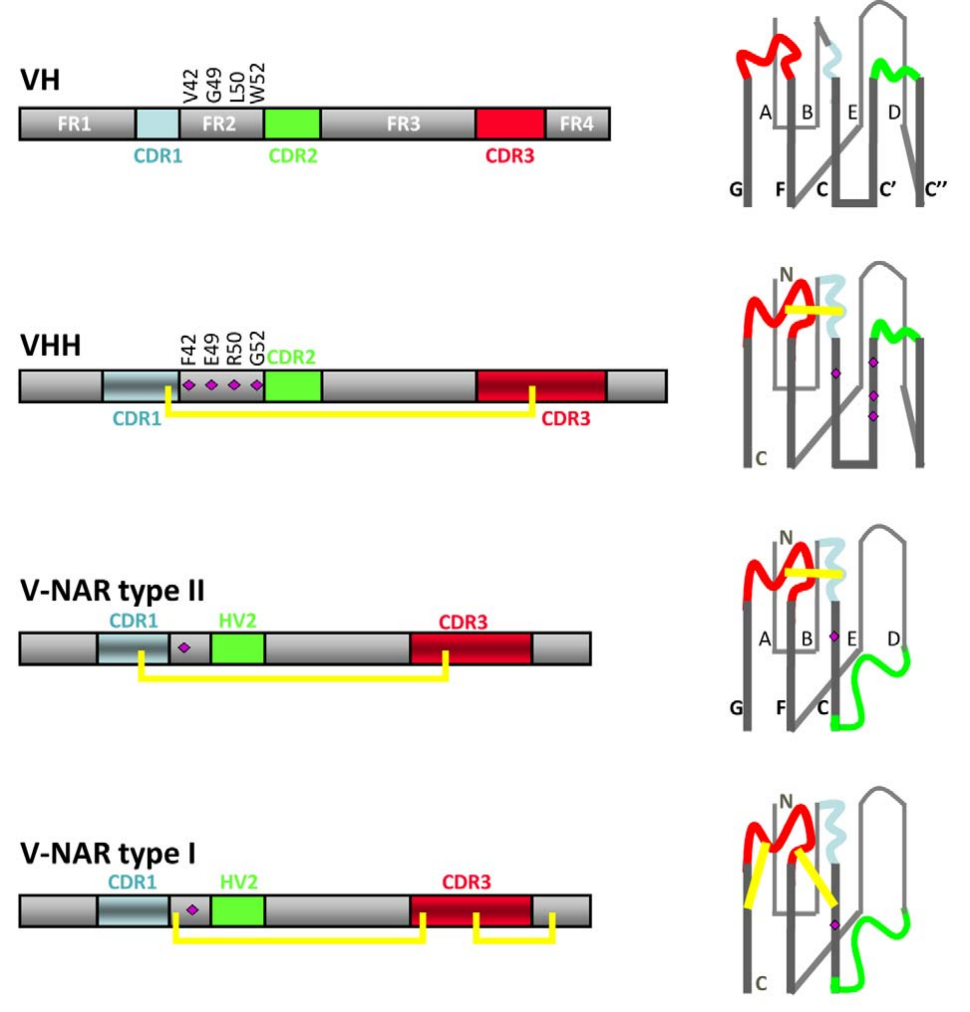
Schematic of the rearranged VH, VHH, and V-NAR type I or type II. At left are displayed the linear sequence hallmarks such as the complementarity determining region (CDR) within the more conserved framework (FR) residues (gray). The disulfide bonds connecting the CDR3 with the CDR1 or FR in a VHH or V-NAR are shown by the yellow line. The folded structures of the V domains with the nine or seven β strands (named A to G in V-NARs, and A to G with insertion of strands C′ and C′′ between C and D for VH and VHH) forming two β-sheets are shown on the right. The purple diamonds on the structure denote the VHH hallmark residues in FR2 or the polar charged residues in the V-NARs. The N- and C-terminal ends of the polypeptides are shown in the structure of VHH and V-NAR type I.

While the identification of the driving force for the emergence of homodimeric H-chain antibodies is conjectural, the rationale for the presence of unique mutations in V-NAR and VHH framework regions (FRs) and the presence of disulfide bonds in the hypervariable loops are easier to envision. Amino acids in the second FR (FR2), the region between the first and second hypervariable (i.e., antigen-binding) loops are very conserved and hydrophobic in conventional multichain antibodies and are used in VH/VL pairing (Figure 3) [21],[22]. Mutations in these FR2 residues from hydrophobic to charged/polar amino acids are required for the V domain to become independent of its VL partner [4],[13]. The generation of noncanonical, intradomain disulfide bonds between the long antigen-binding loops of V-NAR and VHH increases the stability of the domain, which might have been required to cope with the harsh physiological conditions to which these antibodies are exposed [5], i.e., elevated concentrations of urea in shark blood and an arid environment with extreme temperatures for camelids. In addition, the presence of an interloop or a loop-FR disulfide bond probably restricts the conformational flexibility of the antigen-binding loops in the antigen-free V-domain [19]. This conformational loop constraint in the antigen-free antibody will lead to a lower entropic penalty upon antigen binding, where it is expected that the loops are somehow fixed in a unique position within the antibody-antigen complex.

## Conception of HCAbs in Widely Different Contexts

Camels and sharks, two evolutionarily remote species (Figure 2), display extreme molecular convergence of their Igs. Both species have a dichotomous adaptive humoral immune system with both conventional and homodimeric antibodies and share the features described above. However, the camelid homodimeric antibodies were derived from conventional genes of the IgH locus by a relatively recent adaptation [7], while IgNAR of the shark originated from an ancient, undefined evolutionary event. Shark Ig-NARs might display the primordial condition, although it remains enigmatic how the unassociated V regions of a single-chain antibody could evolve into dimeric Vs; perhaps the high salt/urea conditions described above was one driving force in selecting for a dichotomous immune system.

**Figure 4:**
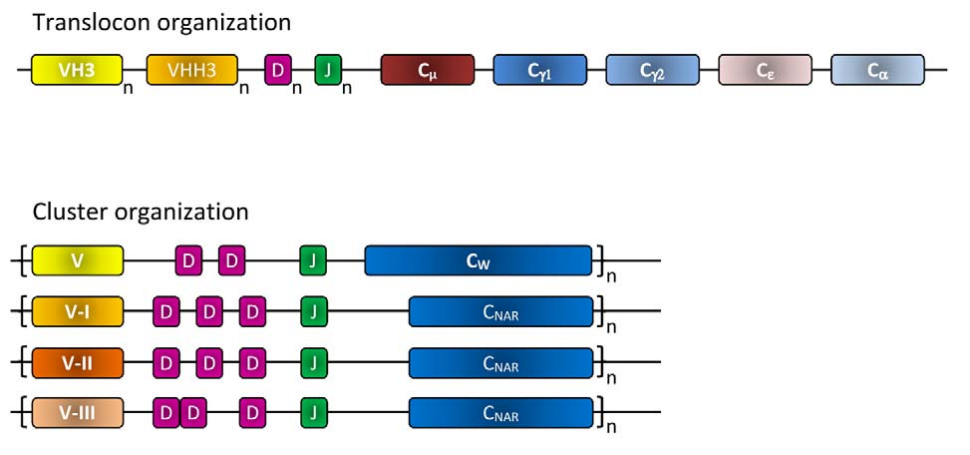
Translocon and cluster genome organization of the antibody genes. The translocon organization (top) of the gene segments as it occurs in camelids to produce the H-chain of classical antibodies and the H-chain of HCAbs. For the former antibody type, one member of the different VH families (shown VH4 and VH3) are rearranged with one of the D and one of the J genes to be transcribed with Cµ and after a class switch with the Cγ1, Cε, or Cα. Note that the greater the number of families and the more members per family, the larger the repertoire will be. For the production of HCAbs, one of the VHH3 genes (or the VH4) has to be rearranged with one of the D and one of the J genes of the same translocon before transcription occurs with the Cµ, and after a class switch, with the Cγ2 (or Cγ3 not shown). To generate the H chain of an H_2_L_2_ antibody, a VH3 gene (or VH4) has to recombine with one D and one J minigene and should be transcribed with the Cµ, or after a class switch with any other C isotype except Cγ2 or Cγ3. Cartilaginous fish have a cluster organization of their antibody genes whereby each cluster contains a dedicated V element followed by two or three D genes and a J gene. After rearrangement of these minigenes, cotranscription occurs with the W constant gene or with the C-NAR genes to produce classical H_2_L_2_ antibodies or HCAbs, respectively. There are multiple clusters in the genome of shark and the V minigenes of some clusters belong to either type I, type II (both clustered with 3-D segments), or type III (clustered with 2-D segments) minigenes.

The question must be addressed: if, indeed, the HCAbs offer a complementary binding site to conventional VH/VL antibodies, why haven’t they been found in all vertebrates? Their presence in all cartilaginous fish, either as a TCR or an Ig [10], is a testament to the unique organization of their antigen receptor genes. While all other vertebrates employ the so-called translocon-type organization (VnDnJnC), cartilaginous fish Ig genes are encoded in clusters (VDJC)n (Figure 4) [10],[11]. Such an organization affords the sharks freedom to evolve new Igs “without much evolutionary fuss,” such as germline-joined clusters for generation of binders of conserved antigens, exchange of genes between TCR and Ig loci, and the single-domain V, HCAbs described here. Indeed, in the holocephalin *Hydrolagus colliei* (spotted ratfish) there has been CH1 loss in an IgM cluster concomitant with a CH2 exon duplication, which likely resulted in the emergence of another homodimeric IgM (Figure 2) [23]; the protein encoded by this gene has not been examined, but it is likely to function as an L-chainless, H-chain dimer, on the basis of the criteria detailed above. Single-domain Vs have evolved also in monotremes and marsupials via generation of an entirely new locus, which is a chimera between Ig and TCR loci (Figure 2) [24]. By contrast, the mechanism by which both camelid HCAbs and conventional antibodies are generated from the same locus during B-cell maturation remains elusive. A novel addition to the classical IgM-IgG class switch must have evolved to allow production of these camelid HCAbs [25]. Thus, HCAbs have emerged by convergence at least four times in vertebrate evolution, and we believe that they are clearly useful and, anthropomorphically speaking, all species would love to have them; however, the organization of Ig and TCR loci in all vertebrates besides cartilaginous fish precludes, or makes quite difficult, the capacity to generate them. Consistent with the idea that single-domain Vs are a useful innovation, once they emerged in the ancestral camelid and cartilaginous fish species, HCAbs have been perpetuated as a major immune system attribute in all of the descendant species.

## Conclusions

We have described the convergent features of single-chain antibodies that permitted their emergence and reemergence and the constraints that may have precluded their existence in all vertebrates. On the basis of recent discoveries, it is possible that they will be found in other species as well, either as an Ig or a TCR. Single-domain Vs have a growing repertoire of uses in both clinical and basic research, a testament to their complement of the conventional VH/VL antibodies; basic research on the animals that have naturally evolved such antibodies will provide refined theories of their dedicated functions, and give greater insight to their emergence several times in vertebrate history.

## Supporting Information

Text S1 Section 1: The rationale for the H_2_L_2_ antibody-based adaptive humoral immune system in jawed vertebrates. Section 2: Absence of CH1 domain in H chains for HCAbs. Section 3: Generation of a large V domain repertoire in absence of the VH-VL combinatorial diversification. Section 4: Producing soluble V domains in absence of VL partner. Section 5: Unique antigen recognition by V-NAR and VHH.
(RTF)

## Abbreviations

C: constant
D: diversity
FR: framework region
H: heavy
HCAb: heavy-chain antibody
Ig: immunoglobulin
J: joining
L: light
NAR: new antigen receptor
TCR: T-cell receptors
VH: variable region of a heavy chain
VL: variable region of a light-chain
VHH: variable region of a heavy chain of a heavy-chain antibody
