# Supplementary material for: A Case Of Convergence: Why Did a Simple Alternative to Canonical Antibodies Arise in Sharks and Camels?
Source: PLoS Biol. 2011 Aug 2;9(8):e1001120. doi: 10.1371/journal.pbio.1001120 (PMC3149040; doi:10.1371/journal.pbio.1001120)
Supplement: Text S1 — Section 1: The rationale for the H2L2 antibody-based adaptive humoral immune system in jawed vertebrates. Section 2: Absence of CH1 domain in H chains for HCAbs. Section 3: Generation of a large V domain repertoire in absence of the VH-VL combinatorial diversification. Section 4: Producing soluble V domains in absence of VL partner. Section 5: Unique antigen recognition by V-NAR and VHH. (RTF) [file pbio.1001120.s001.rtf]

Section 1 
The rationale for the H2L2 antibody-based adaptive humoral immune system in jawed vertebrates
Clearly, the larger and more diverse our primary antibody repertoire, the better and more efficiently we will be protected against infections. The basic concept to produce this seemingly unlimited repertoire of antibodies was discovered for mouse and human decades ago. During B-cell development, one V (variable), one D (diversity) and one J (joining) minigene are selected at will from a pool of gene segments clustered in the IgH locus, and are rearranged to assemble a VH gene that will be co-expressed with a constant (C) gene to produce the H-immunoglobulin polypeptide. Likewise one V and one J minigene are selected from a pool, clustered in an IgL locus, to assemble a VL gene that will be co-expressed with a CL gene to produce an L-chain. Obviously, with greater numbers and more diverse V, D and J elements, larger VH and VL repertoires can be constructed. In addition, the gene-joining process is imprecise, and nucleotides are added or deleted at the joining ends, which increases the possible repertoire of VH and VL domains to well over 106 and 104 possibilities, respectively. To complete expression of the antibody in the B-cell, the H-chain pairs with the L-chain so that the VH and VL are joined at “the front end” of the molecule where they are available for antigen recognition (Figure 1, main text). This VH-VL combination is responsible for the explosion of theoretical antigen-binding sites to well over 1010. 


Section 2
Absence of CH1 domain in H chains for HCAbs
An ingenious but simple mechanism ensures that the H-chains are only released from the B-cells after pairing with an L-chain. An expressed H-chain is sequestered in the endoplasmic reticulum by an Ig-binding protein (BiP) that interacts with the first C domain of the H-chain (CH1 region). To release the H-chain from the endoplasmic reticulum, an L-chain must displace BiP to initiate the secretion of the complete antibody [1,2]. 
Therefore, in absence of the CH1 domain, the H-chain assembles as a homodimeric H molecule that exits the endoplasmic reticulum to become inserted in the membrane or secreted as an HCAb. In camelids, the g genes dedicated for HCAbs acquired a mutation at the canonical splicing site located at the CH1/5' intron border [3,4]. Consequently, this truncated exon/intron boundary is no longer recognized by the spliceosome so that splicing of the mRNA occurs between the VHH exon and the hinge exon, whereby the nucleotides encoding a CH1 domain are deleted and H-chain homodimers are secreted [5].
The lack of a typical CH1 domain clearly occurred via a different mechanism in the shark. It has been speculated that H and L-chain RAG-mediated gene rearrangements occur simultaneously in developing B cells in sharks [6], which suggests that the active IgNAR H-chain might interact with an expressed L-chain. (Note that in mammals the H chain rearrangement occurs in pro-B cells and precedes the L chain rearrangement that occurs in the pre-B cell stage).  Thus, in case of shark IgNAR, the CH1 domain should be modified or absent to prevent L-chain association. As mentioned above, IgNAR is composed of 5 C domains and is a divergent IgH isotype related to a more conventional shark isotype, IgW, which has 6 C domains [7-9]. We hypothesized that early in cartilaginous fish history a VNAR-D-J cluster recombined with an IgW cluster whereby the IgW cluster lost its V-D-J and first C exon, to create the IgNAR isotype [10]. Consequently, the last 4 C domains of IgNAR are clearly homologous to the last 4 domains of IgW, while a homologue of the IgW CH1 domain is absent [11,12]. Phylogenetic analysis by Bernstein et al. [13] suggested that while the NAR C1 domain is divergent from all other C domains in the database, it is somewhat similar to the IgW-CH2 domain and may be derived from this domain, suggesting that all of the IgNAR and IgW C domains are orthologous. Interestingly, in addition to the loss of BiP- and L-chain-interaction sites on the NAR C-1 domain, a unique hinge region evolved at the N-terminus to provide great flexibility of the single V-NAR [7,8].

References
1. Feige MJ, Groscurth S, Marcinowski M, Shimizu Y, Kessler H, et al. (2009) An unfolded CH1 domain controls the assembly and secretion of IgG antibodies. Mol Cell 34: 569-579.
2. Haas IG, Wabl M (1983) Immunoglobulin heavy chain binding protein. Nature 306: 387-389.
3. Nguyen VK, Hamers R, Wyns L, Muyldermans S (1999) Loss of splice consensus signal is responsible for the removal of the entire C(H)1 domain of the functional camel IGG2A heavy-chain antibodies. Mol Immunol 36: 515-524.
4. Woolven BP, Frenken LG, van der Logt P, Nicholls PJ (1999) The structure of the llama heavy chain constant genes reveals a mechanism for heavy-chain antibody formation. Immunogenetics 50: 98-101.
5. Nguyen VK, Zou X, Lauwereys M, Brys L, Bruggemann M, et al. (2003) Heavy-chain only antibodies derived from dromedary are secreted and displayed by mouse B cells. Immunology 109: 93-101.
6. Malecek K, Lee V, Feng W, Huang JL, Flajnik MF, et al. (2008) Immunoglobulin heavy chain exclusion in the shark. PLoS Biol 6: e157.
7. Greenberg AS, Avila D, Hughes M, Hughes A, McKinney EC, et al. (1995) A new antigen receptor gene family that undergoes rearrangement and extensive somatic diversification in sharks. Nature 374: 168-173.
8. Roux KH, Greenberg AS, Greene L, Strelets L, Avila D, et al. (1998) Structural analysis of the nurse shark (new) antigen receptor (NAR): molecular convergence of NAR and unusual mammalian immunoglobulins. Proc Natl Acad Sci U S A 95: 11804-11809.
9. Anderson MK, Strong SJ, Litman RT, Luer CA, Amemiya CT, et al. (1999) A long form of the skate IgX gene exhibits a striking resemblance to the new shark IgW and IgNARC genes. Immunogenetics 49: 56-67.
10. Criscitiello MF, Saltis M, Flajnik MF (2006) An evolutionarily mobile antigen receptor variable region gene: doubly rearranging NAR-TcR genes in sharks. Proc Natl Acad Sci U S A 103: 5036-5041.
11. Greenberg AS, Hughes AL, Guo J, Avila D, McKinney EC, et al. (1996) A novel "chimeric" antibody class in cartilaginous fish: IgM may not be the primordial immunoglobulin. Eur J Immunol 26: 1123-1129.
12. Berstein RM, Schluter SF, Shen S, Marchalonis JJ (1996) A new high molecular weight immunoglobulin class from the carcharhine shark: implications for the properties of the primordial immunoglobulin. Proc Natl Acad Sci U S A 93: 3289-3293.


Section 3
Generation of a large V domain repertoire in absence of the VH-VL combinatorial diversification
Since the success of our adaptive humoral immune response relies largely to its capacity to produce (continuously and in advance) multiple antibody specificities against any possible invader, it is extremely surprising to discover that antibodies of some species have progressed to functional H-chain-only antibodies. This eliminates the combinatorial H-L diversification of conventional, heterotetrameric antibodies, and therefore one would expect a poorly diversified antigen-binding repertoire in these HCAbs. 
As mentioned in the Text Section 1, the first level of antibody diversity originates from the random and imprecise combinatorial joining of V-(D-)J genes. Then this diversity is enhanced by the combinatorial VH-VL domain pairing. Homodimeric antibodies such as HCAbs and IgNAR lack L-chain diversity and the subsequent H-L combinatorial diversification; therefore alternative diversification mechanisms have evolved to compensate for this lack of heterogeneity on HCAbs or IgNARs. 
In sharks, three or four rearrangement events (VDDDJ) generate the CDR3 (Figure 4, main text), resulting in long and heterogeneous binding sites. Furthermore, it was shown through extensive cDNA analysis that the mutation frequency for IgNAR was exceptionally high [1]. This observation of preferential targeting of mutations forced revision of the original hypothesis that the IgNAR mutations generated the primary repertoire [2]; remarkably, the mutations were present mainly in the secretory IgNAR whilst the transmembrane form was practically unmutated. This extensive somatic hypermutation was a surprising finding since sharks, as ectothermic animals that lack germinal centers, were thought previously to lack, or at least not optimally exploit, the process of somatic hypermutation [3]. Subsequently, through affinity maturation studies it became clear that hypermutation of IgNAR is comparable and even more extensive than is found in mammalian immunoglobulins [1,4-6]. More importantly, these studies also showed that there was a strong positive selection (higher replacement/silent mutation ratio) in CDR1 of type-II and the hypervariable loop 2 of type-I V-NAR regions [7,8]. 
Camelid VHHs also display an elevated level of somatic hypermutation. Notable is the higher occurrence of hypermutation hotspots encoded in the VHH germline genes in comparison to the conventional VH germline genes [9]. The VHH genes acquired two new hotspot regions (TAY-codons) upstream of CDR1, which encode key residues for the H1 loop conformation. Somatic variants of the amino acids enlarge the hypervariability in this region (Figure 3, main text), possibly change the loop backbone conformation and affect directly the antigen binding [10,11]. 
Consequently, it seems that both VHH and V-NAR employ somatic hypermutation to an extensive degree, which has a striking impact on antigen recognition since this hypermutation instigates different loop conformations. The absence of L-chain pairing likely provides 'freedom' for higher levels of mutations as well as longer and heterogeneous CDR3 in the single V- domain.
Besides somatic hypermutation, the V-NAR and VHH domains developed an additional strategy to diversify their antigen-binding site. It was noticed that, in contrast to classical VHs, the camelid VHH germline genes encode an extra, non-canonical cysteine within CDR1 (or FR2) (Figure 3, main text). The VHH genes were categorized into subfamilies based on the position of these non-canonical cysteines and the length of the CDR2 [9]. Similarly, the IgNAR V genes also encode non-canonical cysteine residues and based on the different number and location of these cysteines, V-NARs are categorized into type-I and type-II domains (Figure 3, main text). Type-II V-NAR domains have a cysteine in CDR1 which forms an interloop disulfide bond with the cysteine of CDR3 [12], thus stabilizing the loops of the V-NAR domain [13]. Type-I V-NAR domains are similar in overall structure with type-II V-NAR, but they have conserved cysteines in FR2 and FR4 [2,4,8] that form disulfide bonds with cysteines in CDR3 that are generally encoded by the D regions read in a preferred reading frame [4]. Thus the V-NAR type-I uses two loop-FR disulfide bonds between CDR3 and FR2/FR4 [4,12]. In some V-NAR Type I sequences a second set of cysteines are present in CDR3 that are presumed to form an intraloop disulfide bond. A disulfide bond between the CDR3 and the FR2 is also observed among camelid VHH sequences, although at a low frequency. The placement of the disulfide bonds in shark V-NAR profoundly affects the size of the CDR3 (much longer in type I V-NAR) and the differential mutational selection on the CDR, as described above.
The structural analysis of VHHs and V-NAR domains revealed that the differential placement of non-canonical cysteine residues induces very different CDR3 conformations, resulting in remarkably different binding-site topologies [8,13-15]. Thus, these disulfide bonds not only provide stability for the V domains, but also allow generation of a wide variety of geometrical loop architectures which actively contribute to the expansion of the structural repertoire of available paratopes [10,12,16-18]. Therefore, the introduction of non-canonical cysteines forming disulfide bonds between two antigen-binding loops or between the CDR3 loop and the FR is a common strategy among the V-regions of homodimeric antibodies. Thus, the presence and positioning of extra cysteines in the V domains and their oxidation into distinctive intradomain or interloop disulfide bonds in VHH and V-NAR domains exhibit a remarkable convergent molecular evolution.

References
1. Diaz M, Greenberg AS, Flajnik MF (1998) Somatic hypermutation of the new antigen receptor gene (NAR) in the nurse shark does not generate the repertoire: possible role in antigen-driven reactions in the absence of germinal centers. Proc Natl Acad Sci U S A 95: 14343-14348.
2. Greenberg AS, Avila D, Hughes M, Hughes A, McKinney EC, et al. (1995) A new antigen receptor gene family that undergoes rearrangement and extensive somatic diversification in sharks. Nature 374: 168-173.
3. Hinds-Frey KR, Nishikata H, Litman RT, Litman GW (1993) Somatic variation precedes extensive diversification of germline sequences and combinatorial joining in the evolution of immunoglobulin heavy chain diversity. J Exp Med 178: 815-824.
4. Roux KH, Greenberg AS, Greene L, Strelets L, Avila D, et al. (1998) Structural analysis of the nurse shark (new) antigen receptor (NAR): molecular convergence of NAR and unusual mammalian immunoglobulins. Proc Natl Acad Sci U S A 95: 11804-11809.
5. Diaz M, Velez J, Singh M, Cerny J, Flajnik MF (1999) Mutational pattern of the nurse shark antigen receptor gene (NAR) is similar to that of mammalian Ig genes and to spontaneous mutations in evolution: the translesion synthesis model of somatic hypermutation. Int Immunol 11: 825-833.
6. Dooley H, Stanfield RL, Brady RA, Flajnik MF (2006) First molecular and biochemical analysis of in vivo affinity maturation in an ectothermic vertebrate. Proc Natl Acad Sci U S A 103: 1846-1851.
7. Dooley H, Flajnik MF (2006) Antibody repertoire development in cartilaginous fish. Dev Comp Immunol 30: 43-56.
8. Diaz M, Stanfield RL, Greenberg AS, Flajnik MF (2002) Structural analysis, selection, and ontogeny of the shark new antigen receptor (IgNAR): identification of a new locus preferentially expressed in early development. Immunogenetics 54: 501-512.
9. Nguyen VK, Hamers R, Wyns L, Muyldermans S (2000) Camel heavy-chain antibodies: diverse germline V(H)H and specific mechanisms enlarge the antigen-binding repertoire. EMBO J 19: 921-930.
10. Desmyter A, Transue TR, Ghahroudi MA, Thi MH, Poortmans F, et al. (1996) Crystal structure of a camel single-domain VH antibody fragment in complex with lysozyme. Nat Struct Biol 3: 803-811.
11. Muyldermans S, Cambillau C, Wyns L (2001) Recognition of antigens by single-domain antibody fragments: the superfluous luxury of paired domains. Trends Biochem Sci 26: 230-235.
12. Stanfield RL, Dooley H, Flajnik MF, Wilson IA (2004) Crystal structure of a shark single-domain antibody V region in complex with lysozyme. Science 305: 1770-1773.
13. Streltsov VA, Varghese JN, Carmichael JA, Irving RA, Hudson PJ, et al. (2004) Structural evidence for evolution of shark Ig new antigen receptor variable domain antibodies from a cell-surface receptor. Proc Natl Acad Sci U S A 101: 12444-12449.
14. Conrath KE, Wernery U, Muyldermans S, Nguyen VK (2003) Emergence and evolution of functional heavy-chain antibodies in Camelidae. Dev Comp Immunol 27: 87-103.
15. Fennell BJ, Darmanin-Sheehan A, Hufton SE, Calabro V, Wu L, et al. (2010) Dissection of the IgNAR V domain: molecular scanning and orthologue database mining define novel IgNAR hallmarks and affinity maturation mechanisms. J Mol Biol 400: 155-170.
16. De Genst E, Silence K, Decanniere K, Conrath K, Loris R, et al. (2006) Molecular basis for the preferential cleft recognition by dromedary heavy-chain antibodies. Proc Natl Acad Sci U S A 103: 4586-4591.
17. Stanfield RL, Dooley H, Verdino P, Flajnik MF, Wilson IA (2007) Maturation of shark single-domain (IgNAR) antibodies: evidence for induced-fit binding. J Mol Biol 367: 358-372.
18. Streltsov VA, Carmichael JA, Nuttall SD (2005) Structure of a shark IgNAR antibody variable domain and modeling of an early-developmental isotype. Protein Sci 14: 2901-2909.


Section 4
Producing soluble V domains in absence of VL partner
The IgH locus of camelid genome encodes V elements that are dedicated for VHH domain assembly. These so-called VHH germline genes cluster with the human VH3 family in phylogenetic trees [1,2]. However, VHHs are easily distinguishable from VHs due to codon adaptations that resulted in Val42Phe, Gly49Glu, Leu50Arg and Trp52Gly substitutions (IMGT numbering) in the framework-2 region (FR2) of the V domain (Figure 3, main text). These highly conserved and hydrophobic amino acids in VH (Val, Gly, Leu, Trp) are crucial for the association with the VL domain [3], and their hallmark substitution in VHH abrogate such a VL pairing. The hallmark amino acid substitutions in the VHH to more hydrophilic amino acids do not induce any major conformational rearrangement of the backbone [4,5] although they enhance the solubility of the VHH domain [6]. 
Like the camelid VHHs, the shark V-NAR regions display a poor conservation of residues responsible for VH-VL interaction [3,7]. Remarkably, the shark V-NAR genes evolved in such a way that a large portion of the FR2 and the CDR2 is absent, and a single “belt region” called HV2 connects the 2 b-sheets (Figure 3, main text), similar to a C1-type Ig superfamily domain. This reduction of the FR2/CDR2 segments and their replacement by HV2 confer the V-NAR characteristic small size of 12 kDa [8]. In addition to this deletion, the shark V-NAR domains exhibit significant amino acid heterogeneity in its remaining FR2/CDR2 with an increased frequency of charged and polar residues like in VHHs. All of these features reflect the structural plasticity of the V-NAR domain, but more importantly they are responsible for inhibiting VL pairing [7,9-11].


References
1. Muyldermans S, Atarhouch T, Saldanha J, Barbosa JA, Hamers R (1994) Sequence and structure of VH domain from naturally occurring camel heavy chain immunoglobulins lacking light chains. Protein Eng 7: 1129-1135.
2. Vu KB, Ghahroudi MA, Wyns L, Muyldermans S (1997) Comparison of llama VH sequences from conventional and heavy chain antibodies. Mol Immunol 34: 1121-1131.
3. Chothia C, Novotny J, Bruccoleri R, Karplus M (1985) Domain association in immunoglobulin molecules. The packing of variable domains. J Mol Biol 186: 651-663.
4. Desmyter A, Transue TR, Ghahroudi MA, Thi MH, Poortmans F, et al. (1996) Crystal structure of a camel single-domain VH antibody fragment in complex with lysozyme. Nat Struct Biol 3: 803-811.
5. Muyldermans S, Cambillau C, Wyns L (2001) Recognition of antigens by single-domain antibody fragments: the superfluous luxury of paired domains. Trends Biochem Sci 26: 230-235.
6. Davies J, Riechmann L (1994) 'Camelising' human antibody fragments: NMR studies on VH domains. FEBS Lett 339: 285-290.
7. Roux KH, Greenberg AS, Greene L, Strelets L, Avila D, et al. (1998) Structural analysis of the nurse shark (new) antigen receptor (NAR): molecular convergence of NAR and unusual mammalian immunoglobulins. Proc Natl Acad Sci U S A 95: 11804-11809.
8. Greenberg AS, Avila D, Hughes M, Hughes A, McKinney EC, et al. (1995) A new antigen receptor gene family that undergoes rearrangement and extensive somatic diversification in sharks. Nature 374: 168-173.
9. Fennell BJ, Darmanin-Sheehan A, Hufton SE, Calabro V, Wu L, et al. (2010) Dissection of the IgNAR V domain: molecular scanning and orthologue database mining define novel IgNAR hallmarks and affinity maturation mechanisms. J Mol Biol 400: 155-170.
10. Dooley H, Stanfield RL, Brady RA, Flajnik MF (2006) First molecular and biochemical analysis of in vivo affinity maturation in an ectothermic vertebrate. Proc Natl Acad Sci U S A 103: 1846-1851.
11. Streltsov VA, Varghese JN, Carmichael JA, Irving RA, Hudson PJ, et al. (2004) Structural evidence for evolution of shark Ig new antigen receptor variable domain antibodies from a cell-surface receptor. Proc Natl Acad Sci U S A 101: 12444-12449.


Section 5
Unique antigen recognition by V-NAR and VHH
The exact epitope on the commonly used experimental antigen hen egg-white lysozyme (HEL) that is targeted by VHH or V-NAR has been determined by crystallography. In both cases the single V domain recognizes the catalytic site of the enzyme [1-4]. This is surprising as clefts on the antigen surface are not antigenic for classical antibodies that prefer to interact with flat surfaces on the antigen. The shared preference of the single domain V-NAR and VHH for clefts probably results from a required adaptation to increase the size of the interacting surface to a functional level. Indeed, it can be assumed that the absence of the VL (with its 3 antigen binding loops) is compensated in a VHH (and V-NAR) by inserting the protruding antigen binding loops at the end of the prolate (rugby ball-like shaped), single domain particle in the concave shaped surfaces of the antigen to increase the antigen-contacting surface. Since the isolated VHH contain many enzyme active site binders (i.e. act as enzyme inhibitors), it has been theorized that the targeting of clefts or crevices is a cardinal property of the single domain antibody regions [5]. [Of note, the lamprey single domain variable lymphocyte receptor (VLRB.2D) was also shown to interact with the active site of lysozyme, although its antigen binding site has a concave architecture equipped with a protruding loop that inserts into the active site of the antigen [6]. 


References
1. Desmyter A, Transue TR, Ghahroudi MA, Thi MH, Poortmans F, et al. (1996) Crystal structure of a camel single-domain VH antibody fragment in complex with lysozyme. Nat Struct Biol 3: 803-811.
2. Stanfield RL, Dooley H, Flajnik MF, Wilson IA (2004) Crystal structure of a shark single-domain antibody V region in complex with lysozyme. Science 305: 1770-1773.
3. De Genst E, Silence K, Decanniere K, Conrath K, Loris R, et al. (2006) Molecular basis for the preferential cleft recognition by dromedary heavy-chain antibodies. Proc Natl Acad Sci U S A 103: 4586-4591.
4. Stanfield RL, Dooley H, Verdino P, Flajnik MF, Wilson IA (2007) Maturation of shark single-domain (IgNAR) antibodies: evidence for induced-fit binding. J Mol Biol 367: 358-372.
5. Lauwereys M, Arbabi Ghahroudi M, Desmyter A, Kinne J, Holzer W, et al. (1998) Potent enzyme inhibitors derived from dromedary heavy-chain antibodies. EMBO J 17: 3512-3520.
6. Velikovsky CA, Deng L, Tasumi S, Iyer LM, Kerzic MC, et al. (2009) Structure of a lamprey variable lymphocyte receptor in complex with a protein antigen. Nat Struct Mol Biol 16: 725-730.
